# miRNAs as Epigenetic Biomarkers in the Study of the Bidirectional Relationship between Type 2 Diabetes Mellitus and Periodontitis: A Systematic Review

**DOI:** 10.3390/ijms251910723

**Published:** 2024-10-05

**Authors:** María Mata-Monterde, Ana Serrano-Valcarce, Pedro José Almiñana-Pastor, Pablo Micó-Martínez, Andrés López-Roldán

**Affiliations:** Department of Stomatology, Faculty of Medicine and Odontology, University of Valencia, 46010 Valencia, Spain

**Keywords:** periodontitis, diabetes mellitus (DM), miRNAs, epigenetic, gingival crevicular fluid (GCF), oral fluids

## Abstract

The objective of this study is to analyze the miRNA expression of oral fluids such as gingival crevicular fluid (GCF) in patients with periodontitis and Type 2 diabetes mellitus, and how these epigenetic biomarkers can influence the bidirectional relationship of these two inflammatory diseases. This review was conducted following the PRISMA criteria. PubMed, Scopus, Cochrane Library, Embase, and Web of Science databases were searched for clinical studies conducted on humans investigating, through GCF miRNA expression, the relationship between periodontal diseases and type 2 diabetes mellitus. In addition, the etiopathogenic pathways of the studied miRNAs were analyzed using the DIANA MIR path tool. A total of 1436 references were identified in the initial literature search, and seven articles were finally included in this review. Most of the articles included in this review were case–control studies and examined the expression of miRNAs in patients with periodontitis with or without diabetes. Due to their characteristics, miRNAs appear to be the ideal biomarkers for improving the understanding and knowledge of the etiopathogenic pathways that link both diseases. Among all the studied miRNAs, miR-146a, miR-155, miR-200b, miR-223, and miR-203 showed strong involvement in inflammatory and metabolic pathways, making them potential good diagnostic and prognostic biomarkers.

## 1. Introduction

Periodontitis is a multifactorial chronic inflammatory and destructive disease that affects the periodontium, compromising the supporting structures of the teeth (gingiva, cementum, the periodontal ligament, and the alveolar bone) and negatively impacting overall health [1].

A distinctive feature of periodontitis is an exaggerated immune inflammatory response that leads to systemic consequences characterized by an increased inflammatory burden attributed to the persistence of low-grade chronic inflammation over prolonged periods, contributing to numerous systemic disorders [2].

Chronic inflammation is primarily mediated by cytokines, which are generally classified as pro-inflammatory, such as tumor necrosis factor α (TNF-α), or anti-inflammatory, such as interleukin-10 (IL-10); the balance of these mediators reflects the degree of systemic inflammatory burden. TNF-α stimulates the destruction of connective tissue and the alveolar bone by increasing osteoclast activity and promoting the degradation of the extracellular matrix. It also promotes the production of other inflammatory cytokines and factors that perpetuate inflammation, leading to the progression of periodontal disease and contributing to insulin resistance [3].

Therefore, uncontrolled diabetes, characterized by chronic low-grade inflammation, leads to increased production of TNF-α, which worsens periodontal inflammation. At the same time, chronic periodontitis with high TNF-α activity can increase systemic inflammation, further aggravating insulin resistance and making diabetes control more difficult [4].

IL-10, an anti-inflammatory cytokine, attempts to control inflammation, but its effectiveness may be limited in both cases when chronic inflammation remains uncontrolled [5].

Diabetes mellitus (DM) represents a commonly severe metabolic disorder affecting blood glucose levels caused by either insulin secretion deficiency (type 1 diabetes mellitus, DM1) or insulin resistance (type 2 diabetes mellitus, DM2), the latter accounting for approximately 90% of all DM cases worldwide [6].

### 1.1. Bidirectional Relationship between Periodontitis and DM

Recently, scientific evidence has observed a link between periodontal diseases and other general health problems, such as diabetes, adverse pregnancy outcomes, and cardiovascular diseases. Diabetes mellitus represents a risk factor for the development and progression of periodontitis. The risk of periodontitis progression increases approximately threefold in diabetic patients compared to non-diabetic patients. According to some studies, the interrelationship between diabetes and periodontal diseases reflects a mutually reinforcing cycle between oxidative stress and inflammation [7].

The relationship between periodontitis and diabetes could be connected through common immuno-inflammatory factors. These factors are activated in response to the immuno-inflammatory stimuli caused by periodontal pathogens. This connection highlights how periodontal infections can influence the control of diabetes mellitus and vice versa. The adjustment of the inflammatory response could be mediated by epigenetic factors such as miRNAs, which might act as an immunoregulation factor [8].

Concretely, Toll-like receptors (TLRs) are mainly responsible for the modulation of innate immunity. The increased concentrations in glucose and lipopolysaccharide of Gram-negative bacteria activates these receptors, triggering a cascade of reactions that results in greater oxidative stress, the release of reactive oxygen species (ROS), and apoptosis. These products can also reactivate the receptors, which contributes to the maintenance of chronic inflammation. Furthermore, activation of TLR4 in macrophages and β-cells mediates the inflammatory pathway, which leads to β-cell dysfunction (reduced production and secretion of insulin) and its apoptosis [9].

In addition, klotho is a membrane-bound protein that acts as an anti-inflammatory modulator, as klotho negatively regulates nuclear factor kappa activity (NF-kB), reducing pro-inflammatory gene transduction and, consequently, protecting from oxidative stress-induced injury. Decreased expression of klotho has been found to be associated with a variety of diseases, including age-related diseases, inflammatory diseases, and bone metabolism-related diseases, such as diabetes and periodontitis. Different miRNAs regulate klotho gene expression at the transcription levels [10,11].

### 1.2. miRNAs as Biomarkers

miRNAs are a large family of shot non-coding RNAs. They are composed of 17–25 nucleotides. More than 2300 miRNAs have been identified in the human genome. miRNAs can control or regulate gene expression [12]. miRNAs can suppress gene expression, orchestrating a series of processes such as host cell immune responses, including antibacterial, anti-inflammatory, and antioxidant responses [13].

Epigenetics is a scientific field that studies changes in gene expression that do not require or involve changes in DNA sequencing. In other words, epigenetics is not directly related to genetic mutation but to the regulation of the expression of certain genes that allow cellular functions to adapt to the cellular needs of the environment [14].

This field of biology allows us to perform a dynamic analysis of variations in gene expression; therefore, epigenetic markers provide considerable advantages over static genetic measurements. These markers can express susceptibility to a disease at different stages of its progression, something that cannot be measured by genetic markers. Additionally, it may contribute to a better understanding of the association between risk factors and susceptibility to periodontitis [15].

In recent years, some research has focused on the molecular components of gingival crevicular fluid (GCF), observing that epigenetic markers such as miRNAs in GCF are good diagnostic and prognostic biomarkers of periodontal changes [16]. Previous studies have shown dysregulation in certain miRNAs, which are induced in many cell types responding to microbial lipopolysaccharide, stimulating NFκB target genes that encode various mediators of inflammation and are involved in oxidative stress processes [17]. Furthermore, it also has been reported in type 2 diabetes, caused by advanced glycation end products, which encourages apoptosis of osteoblasts and endothelial cells [18].

Some of the limitations of miRNAs as epigenetic markers associated with their use in clinical practice are:

- Specificity: miRNAs are not always specific to a particular disease. Many diseases can share expression profiles of certain miRNAs;
- Interindividual variability: miRNA expression can vary considerably between individuals due to factors such as age, gender, lifestyle habits, and the presence of other diseases;
- Cost: Techniques for the precise detection of miRNAs, such as sequencing and real-time PCR, require expensive equipment and specialized personnel [19].

### 1.3. GCF and Saliva as Mediums for miRNAs

The GCF is a transudate released in the gingival sulcus of the teeth. It is primarily composed of polymorphonuclear leukocytes (PMN), serum proteins, bacteria, tissue breakdown products, enzymes, antibodies, and numerous inflammatory mediators and nucleic acids. GCF collection is a simple and non-invasive procedure and is especially useful for identifying individuals at risk for the onset or progression of periodontitis and for monitoring the response to periodontal therapy [20].

Saliva samples or liquid biopsy samples have been shown to be useful as minimally invasive tools. They are essential for molecular investigations in oral disease studies, such as circulating tumor DNA (ctDNA), miRNAs, proteins, and exosomes. Saliva, as an oral fluid, is necessary for oral biofilm formation and host defense, and has been used to evaluate the progression of periodontitis and as a non-invasive diagnostic tool for insulin resistance. Therefore, both saliva and GCF samples are considered important liquid biopsy samples for the analysis of diseases, such as periodontal disease [16]. Some advantages of GCF include local specificity, as it originates in the gingival sulcus, and therefore directly at the sites of periodontal inflammation, allowing for a more accurate measurement of the sites of localized changes in the disease. The high concentrations of inflammatory biomarkers and miRNAs specific to periodontal pathology make GCF a valuable sample. However, it presents some disadvantages, such as its technically complex collection process and its reduced volume compared to a saliva sample. The latter is easier to collect, making it less invasive and more convenient for the patient; however, it is less specific [21,22].

Gingival tissue samples or biopsies serve as useful tools to assess miRNA expression levels, providing a direct sample of the affected site and allowing accurate and specific assessment of the molecular changes in the disease. However, obtaining gingival tissue samples requires an invasive procedure (biopsy), which carries potential complications such as risk of infection, bleeding, and postoperative pain [23].

Despite the numerous studies that have identified epigenetic disorders in various inflammatory diseases and given the recent and novel importance of epigenetics in periodontal diseases, there are few studies that analyze the importance of miRNAs in the bidirectional relationship between periodontitis and diabetes mellitus.

The objective of this systematic review, therefore, is to determine how the relationship between chronic periodontitis and diabetes mellitus is manifested through the expression of miRNAs collected in the oral fluids of adult patients with chronic periodontitis, both with and without diabetes mellitus.

## 2. Materials and Methods

### Study Selection

This systematic review was carried out following the PRISMA (Preferred Reporting Items for Systematic reviews and Meta-Analyses) criteria to identify all the studies that would answer the PECO question (P (Patients/Problem); E (Exposure); C (Comparison); O (Outcome)). A search of the existing literature was performed, using the following electronic databases: PubMed, Embase, Cochrane, Scopus, and Web of Science. No language and publication date filters were applied to avoid the loss of potentially relevant articles. The search for the articles began in February 2024 and concluded in March 2024. The review was registered in PROSPERO with the ID 573972.

The PECO question was formulated as follows:

How does the presence of periodontitis and diabetes mellitus affect the expression of miRNAs in the oral fluids of adult patients?

- P (Patient/Population): adult patients;
- E (Exposition): periodontitis;
- C (Comparison): periodontitis with DM (diabetes mellitus), periodontitis without DM, DM without periodontitis;
- O (Outcome): differential expression of miRNAs in oral fluids.

The inclusion criteria were as follows: randomized clinical trials, case–control studies, cohort studies, and studies addressing the PECO question.

The exclusion criteria were as follows: case report, systematic reviews, and expert opinions; experimental studies using an in vitro sample; and experimental studies conducted on animals.

The search equation used was as follows: ((miRNAs) OR (microRNA) OR (miR) OR (non-coding RNAs) OR (microRNAs) OR (miRNA) OR (epigenetics) OR (epigenetic) OR (epigenome)) AND ((periodontitis) OR (periodontal diseases) OR (periodont*)) AND ((diabetes mellitus) OR (diabetes) OR (systemic diseases) OR (metabolic syndrome)).

The search equation was adapted to the different databases Pubmed, Scopus, Embase, Web of Science, and Cochrane.

Mendeley software 1.19.8 was used to manage the articles found. After discarding duplicate articles from the different databases, the selection process continued by reading the titles and abstracts of the articles found. This process was carried out by two reviewers (PA and MM).

When the title and abstract did not provide enough information, the full text was read. After the first phase of selection, the full texts of the selected articles were read and, in the case of further exclusions, the reasons for rejection were recorded.

The following data were extracted from each of the included studies: author, year of publication, type of study, N (sample size), type of diabetes mellitus (DM1/DM2), type of periodontal disease, sample analyzed, type of epigenetic marker (micro-RNA), results, ROC curve analysis, *p*-value, and miRNA expression levels.

The Newcastle–Ottawa Quality Assessment Scale (NOS) was used to evaluate the quality of the selected articles (Table 1).

## 3. Results

The initial total number of articles found was **1436,** among which there were **2251** duplicates with the same DOI. After discarding duplicate articles from the different databases, the selection process continued with the articles found in the electronic databases by reading the title and abstract. This was performed by two reviewers (PA and MM).

The total number of articles selected was 138. After excluding systematic reviews, the number of articles of interest was reduced to 36 articles. Ten articles were excluded for conducting in vitro experimental studies. Additionally, twelve articles were excluded for conducting in vitro experimental studies on animals. Finally, seven articles were excluded for other reasons considered as grounds for exclusion.

**Figure 1:**
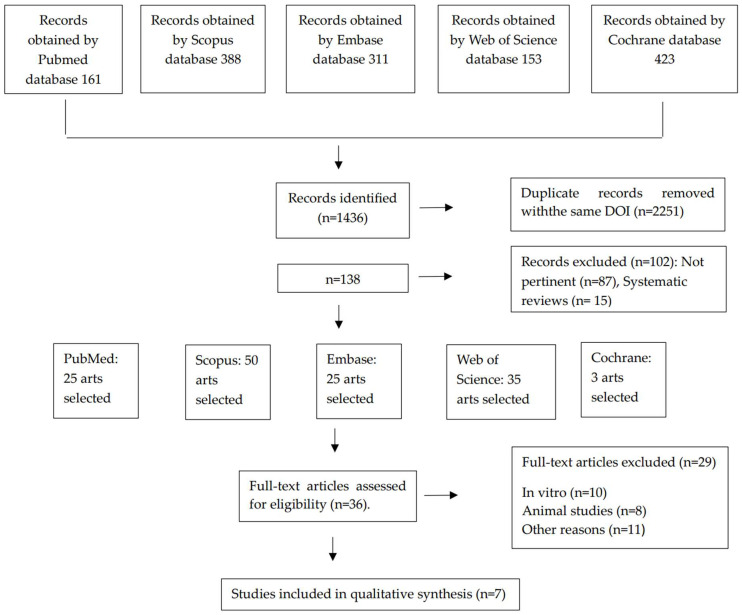
The PRISMA flow diagram summarizes the article selection process.

The final number of articles for this systematic review was seven articles (Figure 1).

### 3.1. Main Results of Included Studies

#### 3.1.1. miR-146a

Radovic et al. (2018) found that miR-146a levels were significantly higher in patients with chronic periodontitis (CP) and CP with type 2 diabetes mellitus (CPDM2) compared to healthy individuals. Additionally, miR-146a was elevated in diabetic patients without periodontal disease compared to periodontally healthy, non-diabetic individuals [24]. Al-Rawi et al. (2020) reported that miR-146a was the most reliable predictor of CP among diabetic subjects and highlighted miR-146a in saliva as a good non-invasive diagnostic biomarker [8].

#### 3.1.2. miR-155

Radovic et al. (2018) also observed significantly higher levels of miR-155 in patients with CP and CPDM2 [24]. Al-Rawi et al. (2020) identified miR-155 as the most reliable predictor of CP among non-diabetic individuals [8]. Bachtiar et al. (2023) noted that miR-155 expression was over five-times higher in patients with diabetes and periodontitis compared to those with periodontitis alone. Additionally, salivary exosomal miR-155 expression levels showed over fivefold upregulation in diabetic patients compared to non-diabetics [25].

#### 3.1.3. miR-203

Al-Rawi et al. (2020) reported that miR-203 was significantly elevated in the saliva of diabetic patients with CP compared to healthy diabetics [8]. Elazazy et al. (2021) found that miR-203 was significantly lower in both the serum and gingival crevicular fluid (GCF) of all studied patients with CP, with or without DM2, compared to controls [26].

#### 3.1.4. miR-223

Elazazy et al. (2021) found that miR-223 was significantly overexpressed in both the serum and GCF of patients with CP, with or without DM2, with higher expression levels in the serum [26]. Liu (2022) observed significantly higher levels of miR-223 in the periodontal group compared to healthy controls, with a lower expression in DM2 compared to CP, but an increased expression in the comorbidity group [15]. Sawangpanyangkura and Teerat et al. (2022) reported the highest expression of miR-223 in GCF among women with gestational diabetes mellitus (GDM) and periodontitis. They also noted a positive regulation of miR-223 expression in GCF but a negative expression in peripheral blood (PB), with a negative correlation between GCF and PB expression, although no significant differences were found [27].

#### 3.1.5. miR-200b

Elazazy et al. (2021) found that miR-200b was significantly overexpressed in both the serum and GCF of patients with CP, with or without DM2, with higher expression levels in the serum [26]. Lui (2022) reported that miR-200b levels were significantly higher in the periodontal group compared to healthy controls, with a lower expression in DM2 compared to CP, but an increased expression in the comorbidity group [15].

#### 3.1.6. miR-106 and miR-103

Lui (2022) observed that miR-106 and miR-103 levels in the GCF were significantly higher in the periodontal group compared to healthy controls, although the increases were lower than those of miR-223 and miR-200b [15].

#### 3.1.7. miR-25-3p

Jing Ni et al. (2023) found a higher salivary expression of miR-25-3p in patients with osteoporosis (OP) and T2DM who also had periodontitis compared to those with a healthy periodontium. There was also a higher salivary expression of miR-25-3p in patients with DM2 compared to healthy individuals, with the T2DM/OP + PD group showing increased salivary expression, indicating changes due to the presence of periodontal disease among patients with OP/T2DM [16].

### 3.2. Characteristics of Included Studies

The characteristics of the studies included in the review are summarized in Table 2 and Table 3. The seven selected articles were cross-sectional observational case–control studies. Sample sizes ranged from 29 to 248 subjects. Regarding the type of diabetes mellitus analyzed, six articles examined type 2 diabetes mellitus (T2DM), one article did not specify the type of diabetes, and one article analyzed gestational diabetes mellitus (GDM). All seven articles analyzed miRNAs as an epigenetic mechanism. The sample type used to investigate the miRNAs was unstimulated saliva in three articles and gingival crevicular fluid (GCF) in four articles. Additionally, three of the studies that analyzed GCF also examined the expression of miRNAs in the serum. Of the selected articles, the miRNAs that appear repeatedly in the different articles are the following: 146, 203, 155, 223, 200b, 103, 106, and 25.

## 4. Discussion

miRNAs play critical roles in various immune processes, and affect both the innate and humoral responses of the host. The current systematic review examined the pattern of miRNA changes in periodontitis associated with diabetes mellitus, since they may aggravate each other’s disease burden, and to determine if these epigenetic markers can be considered as diagnostic or prognostic disease biomarkers.

Sample sizes differed greatly among the studies, obtained from different biological sources, and some authors focused on specific miRNAs. As a result of these methodological differences among studies, we were not able to conduct a meta-analysis. For example, the sample size of the selected studies ranged from 90 subjects in the study by Bachtiar et al. (2023) [25] to 187 subjects in the study by Ni et al. (2023) [16]. GCF and saliva were chosen as diagnostic methods because they are simple, easily obtainable, and non-invasive diagnostic and prognostic tools, criteria that gingival biopsies do not meet, which is the reason why they were excluded from this systematic review. Furthermore, the lack of consideration of obesity in most studies could be another limitation, as this condition could act as a modified factor since it seems to further increase the expression of miRNA species [12]. In contrast, the selected studies were cross-sectional case–control studies; these are valuable due to their efficiency and ability to generate more rigorous results.

The majority of these studies focused on identifying the most relevant difference in miRNA expression in healthy and periodontitis-affected gingival tissue with and without type 2 diabetes mellitus. Among these, miR-146a, miR-155, miR-200b, miR-223, and miR-203 appear to be the most relevant reliable and non-invasive diagnostic and prognostic biomarkers.

Radovic et al. (2018) compared the expression levels of miR-146a and miR-155 in gingival crevicular fluid before and after non-surgical periodontal treatment in patients with chronic periodontitis with and without type 2 diabetes mellitus. They observed significantly higher levels of both miRNAs in patients with chronic periodontitis without diabetes and patients with chronic periodontitis with diabetes. Furthermore, they observed a significant reduction in the expression of both miRNAs after non-surgical periodontal treatment [24]. These two miRNAs seem to have similar function as both are related to the activation of TLR receptors [28].

Al-Rawi et al. (2020) investigated the expression levels of miR-146a, miR-146b, miR-155, and MiR-203 in unstimulated saliva of patients with chronic periodontitis with and without diabetes mellitus. They found that all four miRNAs were overexpressed in patients with periodontitis, especially in those with diabetes. The highest effect was observed for miR-146b and miR-155, in agreement with previous findings. However, they observed that none of the miRNAs could function as potential predictive biomarkers for diabetes among patients with periodontitis. In addition, among the four miRNAs studied, miR-146b was the least reliable predictor in the different study groups [8].

Elazazy et al. (2021) and Lui L et al. (2022) both compared miRNA expression levels in the serum and gingival crevicular fluid in patients with chronic periodontitis (stage II) and type 2 diabetes mellitus. Elazazy et al. focused on miR-200b, miR-203, and miR-223 while Lui L et al., in addition to these miRNAs, studied miR-106 and miR-103. In both studies, a higher expression of miRNAs was observed in patients with periodontitis and diabetes mellitus, with significant increases noted in miR-200b and miR-223 expression, both in the gingival crevicular fluid and serum. Elazazy et al. reported a higher area under the curve (AUC) value in the serum. However, Lui L et al. found that miRNA levels in gingival crevicular fluid were significantly higher than those in corresponding serum samples. The expression of miR-203 was significantly lower in the gingival crevicular fluid and serum of all studied patients compared to controls; this occurred in both studies. Lui L et al. also observed increased levels of miR-106 and miR-103, although their expression was lower compared to miR-200b and miR-223 [15,26].

Jing Ni et al. (2023) evaluated the expression level of miR-25-3p in unstimulated saliva for the detection of osteoporosis and periodontitis in comparison to a mixed group of patients with type 2 diabetes (DM2). The salivary expression of miR-25-3p was higher in the groups of patients with diabetes osteoporosis with and without periodontitis compared to groups with healthy periodontium and diabetes. Patients in the type 2 diabetic osteoporosis and periodontitis group exhibited a higher salivary expression. The analysis of the area under the curve (AUC) showed that this biomarker is effective for diagnosing periodontitis, osteoporosis, and/or type 2 diabetes. This suggests that changes in the expression of the biomarker miR-25 in saliva are induced by type 2 diabetes, and these changes become more evident when periodontitis and osteoporosis are also present [16].

Sawangpanyangkura et al. (2022) analyzed the variations in the expression levels of miR-223 in gingival crevicular fluid (GCF) and peripheral blood in pregnant women with gestational diabetes mellitus and/or periodontitis. They observed a relatively higher and statistically significant expression of miR-223 in the GCF and peripheral blood in the group of women with gestational diabetes and periodontitis compared to the other groups. An increased expression was observed, although with a downward trend in peripheral blood compared to gingival crevicular fluid. This difference was not significant, similar to the studies carried out by Elazazy et al. and Lui L et al. The group of women with gestational diabetes and periodontitis exhibited the highest levels of all periodontal variables [27].

Bachtiar et al. (2023) compared the expression levels of miR-155 in unstimulated saliva of patients affected by COVID-19 and periodontitis, with and without diabetes mellitus. The most notable result was an upregulated expression (>5 times) in patients with diabetes compared to patients without diabetes [25].

Therefore, we observe that in all studies included in this systematic review, diabetes and periodontitis are inflammatory diseases that affect miRNA expression, and their effect can be cumulative. These findings highlight the dysregulation of specific miRNAs in patients with chronic periodontitis and type 2 diabetes mellitus, suggesting their potential role as biomarkers for possible diagnostic, disease progression, and treatment responses.

In addition, to better understand the beneficial (or detrimental) effects of each miRNA in these two inflammatory diseases, we performed an analysis of the biological pathways of the nine miRNAs identified in the selected studies of this systematic review, using the DIANA-miRPath version 3 toll (with the DIANA-tarbase algorithm activated). The biological pathways that were finally chosen were involved in processes related to bone and cellular metabolism, inflammatory response, bacterial invasion, immune response, and epithelial adhesion. This indicates that miRNAs were related to the etiopathogenic processes of periodontitis.

The following KEGG pathways were identified: (1) TGF-Beta signaling pathway, (2) HIF-1 signaling pathway, (3) NOD-like receptor signaling pathway, (4) ErB signaling pathway, (5) PI3K-Akt signaling pathway, (6) cell cycle, (7) RIG-I-like receptor signaling pathway, (8) neutrophin signaling pathway, (9) Chagas disease, (10) apoptosis, (11) NF-kappa B signaling pathway, (12) Toll-like receptor signaling pathway, and (13) HTLV-I infection. Chagas disease was included as previous studies have shown that it could influence at the gingival level due to one of its symptoms being gingivitis.

### miARNSs Introduced into the DIANA-miRPath Tool

- Mir146b-3p
- Mir146a-5p
- Mir155-3p
- Mir-203
- Mir-223-5p
- Mir-200b-3p
- Mir-106a-3p
- Mir-103a-3p
- Mir-25

It has been observed that high levels of Hsa-Mir146a-5p decrease TNF-a, IL-1B, and IL-6, thus altering the RANK/RANKL/OPG axis and therefore promoting osteoclastogenesis. Additionally, high levels of miR-146a-5p could activate the transcription factor NF-kB, ultimately leading to the release of proinflammatory molecules. This miR-146a-5p has also been associated with low levels of EGF and TGF-B, which decrease regeneration potential and may be related to unsatisfactory responses to treatment. Therefore, miR-146a-5p could be considered a biomarker of inflammatory response in periodontitis and a risk factor in diabetes mellitus. miR-146a-5p appears to be involved in different biological functions, including regulation of immune response, inflammation, cell development, and differentiation, among others. This appears to play a significant role in both diabetes and periodontitis by regulating the inflammatory response and the function of the cells involved in these diseases. Its dysfunction or dysregulation may contribute to the progression and severity of both conditions [29].

Further research is needed to fully understand the role of miRNAs and their involvement in in periodontal health and disease progression and how they can specifically influence the bidirectional relationship between periodontitis and diabetes mellitus. More well-designed studies are needed to improve the research on these biomarkers. Also, conducting a massive screening of all of them in gingival crevicular fluid is needed, rather than relying on previous studies conducted on gingival biopsies.

## 5. Conclusions

In this study of the bidirectional relationship between type 2 diabetes mellitus (DM2) and periodontitis, epigenetic markers such as miRNAs, due to their characteristics, seem to be ideal biomarkers to improve the understanding and knowledge of the etiopathogenic pathways that link both diseases.

## Figures and Tables

**Table 1:** Quality assessment of observational studies based on the Newcastle–Ottawa Quality Assessment Scale (NOS).

	Selection				Comparability	Exposure			Total
	Case Definition *	Representativeness of the Cases *	Selection of Controls *	Definition of Controls *	Comparability of Cases and Controls on the Basis of the Design or Analysis **	Ascertainment of Exposure *	Same Method of Ascertainment for Cases and Controls *	No-Response Rate *	
[8]		*	*	*	**		*	*	7
[10]		*	*	*	**		*	*	7
[11]		*	*		*	*	*	*	6
[24]		*	*	*	*		*	*	6
[25]		*	*	*	**	*		*	7
[26]	*	*	*		**	*	*	*	8
[27]		*	*	*	*	*		*	6

**Table 2:** Characteristics and main results of included studies.

	Author	Year	Type of Study	N	Type of DM	Periodontal Diseases	Sample	miRNAs	ROC Curve	P	Expression Levels
1	Radovic N [24]	2018	Case–control	96	DM2	CP	GCF	**mi-RNA (miR-146a/155)**	Both miRNAs showed their highest precision for CP = AUC ≥ 0.9; **miR-146a** showed the best performance, with 100% sensitivity and 96% specificity in CP without diabetes.	**miR-146** = positive correlation with PPD in patients with P CPDM2 and CP (Spearman *r* = 0.676 *yr* = 0.641, respectively, *p* < 0.01). **miR-155** shows a significant positive correlation with PPD (Spearman’s *r* = 0.623 and *r* = 0.645, *p* < 0.01).	**miR-146a** (CP) = 4.11; **miR-146a** (CPDM) = 5.45; **miR-155** (CP) = 2.70; **miR-155** (CPDM) = 3.56.
2	Al-Rawi N [8]	2020	Case–control	35	DM	CP	Saliva	**mi-RNA (146a/146b/155/203)**	**miR-203** accuracy of 76.8%; **miR-155** accuracy of 89.3%; **miR-146a** accuracy of 86.6%; **miR-146b** accuracy of 83%.	Among the subjects with CP and DM in comparison with healthy subjects with CP, there was an increase in miRNAs, but this increase was not statistically significant (*p* > 0.05).	**miR-203** = optimal cutoff value was a Ct standardized < 7.66; **miR-155** = Ct value standardized < 8.95; **miR-146a** = Ct value standardized < 11.3; **miR-146b** = Ct value standardized < 9.34.
3	Elazazy O [26]	2021	Case–control	60	DM2	CP	Serum + GCF	**mi-RNA (miR-223, miR-203, miR-200b)**	In serum **miR-223** = significant AUC (0.87) + sensitivity 88% and specificity 73%; **miR-200b** = significant AUC (0.71) + sensitivity 63% and specificity 79%; **miR-203** showed less expression.	**Serum miR-223** (*p* = 0.00031); **serum miR-200b** (*p* = 0.046); **miR-223** = positive correlation with CAL and PPD in both patient groups and with TNF-α (*p* < 0.05) in the CP group; **miR-200b** = positive correlation with CAL, PPD, and TNF-α (*p* < 0.05) and negative with IL-10 in CP; **miR-203** = negative correlation with TNF-α (*p* < 0.05) in both groups.	
4	Liu L [15]	2022	Case–control	97	DM2	Periodontal disease	Serum + GCF	mi-RNA (miR-223, miR-203, miR-200b, miR-106, miR-103)	Serum **miR-223** = sensitivity 60.6% and specificity 46.7%; AUC (0.643). **miR-200b** = sensitivity 51.5% and specificity 92%; AUC (0.744). GCF: **miR-223** = sensitivity 72.7% and specificity 89.3%; AUC (0.862). **miR-200b** = sensitivity 75.58% and specificity 88.87%; AUC (0.880).	Serum **miR-223** (*p* = 0.056) and **miR-200b** (*p* = 0.06). GCF: **miR-223** (*p* < 0.001) and **miR-200b** (*p* = 0.005).	
5	Jing Ni [16]	2023	Case–control	187	DM2	CP	Saliva	miR-25	**miR-25-3p** used as a test to predict a diagnosis of periodontitis in patients with osteoporosis/DM2 = AUC (0.859) (sensitivity: 77.5%; specificity: 84%). miR-25-3p used to predict a diagnosis of DM2 among healthy individuals = AUC (0.886) (sensitivity: 77.78%; specificity: 90.38%).	Among patients with OP/DM2 and periodontitis, salivary expression of miR-25-3p increased as the values of PPD and CAL of the patients improved (*p* < 0.001). It did not show correlation with other periodontal indicators (*p* > 0.05).	
6	Sawangpanyangkura Teerat [27]	2022	Pilot cross-sectional study	40	DMG	CP	GCF + blood	**mi-RNA (miR-223)**		**miR-223** showed a significant difference between the GDM/P and GDM/NP groups (*p* = 0.04). miR-223 in GCF was positively correlated with periodontal parameters, including %PI, %BOP, and mean PD (*p* < 0.001, *p* < 0.001, *p* = 0.006, respectively).	Relative expression levels of miR-223 in GCF: In GDM/P: 2.40; In GDM/NP: 0.69; In NGDM/P: 1.58; In NGDM/NP: 1.00.
7	Bachtiar BM [25]	2023	Case–control	29	DM2	Moderate-to-severe periodontitis	Saliva	**mi-RNA** **(miR-155)**	For miR-155 and TLR-4, (AUC) of predicted periodontitis = 0.83 + sensitivity 80% and specificity 100% for G1, and (AUC) = 0.88 + 90% sensitivity and 100% specificity for G2.	miR-155: same expression between G1 and G2 (*p* < 0.05).	In G1, salivary miR-155 expression level showed a significant positive correlation with TLR-4 mi-RNA expression (*r* = 0.77, *p* = 0.01), and in G2, a significant negative correlation with TLR-4 mi-RNA expression (*r* = −0.70, *p* = 0.009).

DM2 (Type 2 diabetes mellitus); CP (chronic periodontitis); GDM (gestational diabetes mellitus); PPD (probing pocket depth); CAL (clinical attachment level); OP (osteoporosis).

**Table 3:** KEGG pathways included.

KEGG Pathway	Genes	miRNAs	Biological Process
TFG-beta signaling pathway	5	3	Immune R/Tissue homeostasis
HIF-1 signaling pathway	7	3	Pathological/physiological situations of hypoxia. Angiogenesis/Inflammatory P/Bone metabolism
NOD-like receptor signaling pathway	5	3	Immune R/Inflammatory P
ErbB signaling pathway	6	4	Epithelial adhesion/Inflammatory P
PI3K-Akt signaling pathway	17	6	Cellular metabolism/Inflammatory P
Cell cycle	10	5	Inflammatory P/Cellular metabolism
RIG-I-like receptor signaling pathway	7	2	Inflammatory P
Neutrophin signaling pathway	9	5	Nervous system and neuronal development/Metabolism and function
Chagas disease	8	2	Parasitic disease/Inflammatory P
Apoptosis	8	3	Immune R/Inflammatory P
NF-kappa B signaling pathway	8	2	Immune R/Inflammatory P
Toll-like receptor signaling pathway	10	2	Immune R/Inflammatory P
HTLV-I infection	11	5	Inflammatory P/Viral infection

## Data Availability

No data available.
